# Chronic Disease Prediction Using the Common Data Model: Development Study

**DOI:** 10.2196/41030

**Published:** 2022-12-22

**Authors:** Chanjung Lee, Brian Jo, Hyunki Woo, Yoori Im, Rae Woong Park, ChulHyoung Park

**Affiliations:** 1 Evidnet Seongnam Republic of Korea; 2 Department of Biomedical Informatics Ajou University Hospital Suwon Republic of Korea

**Keywords:** common data model, chronic disease, prediction model, machine learning, disease management, data model, disease prediction, prediction, risk prediction, risk factors, health risk

## Abstract

**Background:**

Chronic disease management is a major health issue worldwide. With the paradigm shift to preventive medicine, disease prediction modeling using machine learning is gaining importance for precise and accurate medical judgement.

**Objective:**

This study aimed to develop high-performance prediction models for 4 chronic diseases using the common data model (CDM) and machine learning and to confirm the possibility for the extension of the proposed models.

**Methods:**

In this study, 4 major chronic diseases—namely, diabetes, hypertension, hyperlipidemia, and cardiovascular disease—were selected, and a model for predicting their occurrence within 10 years was developed. For model development, the Atlas analysis tool was used to define the chronic disease to be predicted, and data were extracted from the CDM according to the defined conditions. A model for predicting each disease was built with 4 algorithms verified in previous studies, and the performance was compared after applying a grid search.

**Results:**

For the prediction of each disease, we applied 4 algorithms (logistic regression, gradient boosting, random forest, and extreme gradient boosting), and all models show greater than 80% accuracy. As compared to the optimized model’s performance, extreme gradient boosting presented the highest predictive performance for the 4 diseases (diabetes, hypertension, hyperlipidemia, and cardiovascular disease) with 80% or greater and from 0.84 to 0.93 in area under the curve standards.

**Conclusions:**

This study demonstrates the possibility for the preemptive management of chronic diseases by predicting the occurrence of chronic diseases using the CDM and machine learning. With these models, the risk of developing major chronic diseases within 10 years can be demonstrated by identifying health risk factors using our chronic disease prediction machine learning model developed with the real-world data–based CDM and National Health Insurance Corporation examination data that individuals can easily obtain.

## Introduction

World Health Organization’s Global Action Plan (2013-2020) for noninfectious diseases aims to reduce the premature death rate stemming from chronic diseases by 25% by 2025 [1]. The plan also urges the establishment of national policies and management of performance indicators.

Accordingly, the Ministry of Health and Welfare of South Korea has designated cardiovascular disease, diabetes, chronic respiratory disease, and cancer as chronic diseases to be managed by the government [2] and established a chronic disease management system centered on local hospitals. In March 2014, a community primary care pilot project for high blood pressure and patients with diabetes was initiated. In September 2016, the chronic disease management pilot project was carried out. From January 2019 to the present, a primary medical chronic disease management pilot project was conducted. Nevertheless, chronic diseases remain the primary cause of mortality and increasing medical expenses. According to the Korea Centers for Disease Control and Prevention, in 2020, chronic diseases were responsible for 7 out of 10 deaths in the country, accounting for 83.7% of total medical expenses [3].

Chronic diseases develop from metabolic syndrome that are caused by lifestyle or individual genetic and environmental factors [4]. The development of chronic disease leads to various complications or requires long-term treatment [5]. Therefore, it is important to take preemptive measures along with the prevention of metabolic syndrome. In this respect, it is necessary to develop various disease prediction models to reduce the risk of complications and medical costs.

Fortunately, early-stage disease prediction is gaining momentum with the use of real-world data combined with machine learning technology. Lee et al [6] predicted the risk of metabolic syndrome (area under the curve [AUC]=0.879) using machine learning techniques, and Choi et al [7] predicted disease occurrence using recurrent neural networks (diagnosis up to 79%). Lipton et al [8] predicted the probability of chronic disease by applying long short-term memory (AUC=0.81-0.99). However, the disease occurrence prediction models developed in South Korea used traditional statistical techniques, and most international predictive models were developed for Western White populations and therefore have reduced applicability to other countries and racial groups.

Although there are already many studies using electronic medical record (EMR) and machine learning, they have limitations in requiring a definition of medical terms or preprocessing for standardization in multicentered studies, entailing that these studies cannot be synchronized with other prediction models. There are relatively few papers on predictive model development using the common data model (CDM; although version 6.0 was release recently, version 5.4 of the Observational Medical Outcomes Partnership CDM is supported by the Observational Health Data Sciences and Informatics suite of tools and methods), which can overcome these limitations. In this paper, we aimed to develop a scalable chronic disease prediction model using the CDM.

## Methods

### Subjects

**Figure 1:**
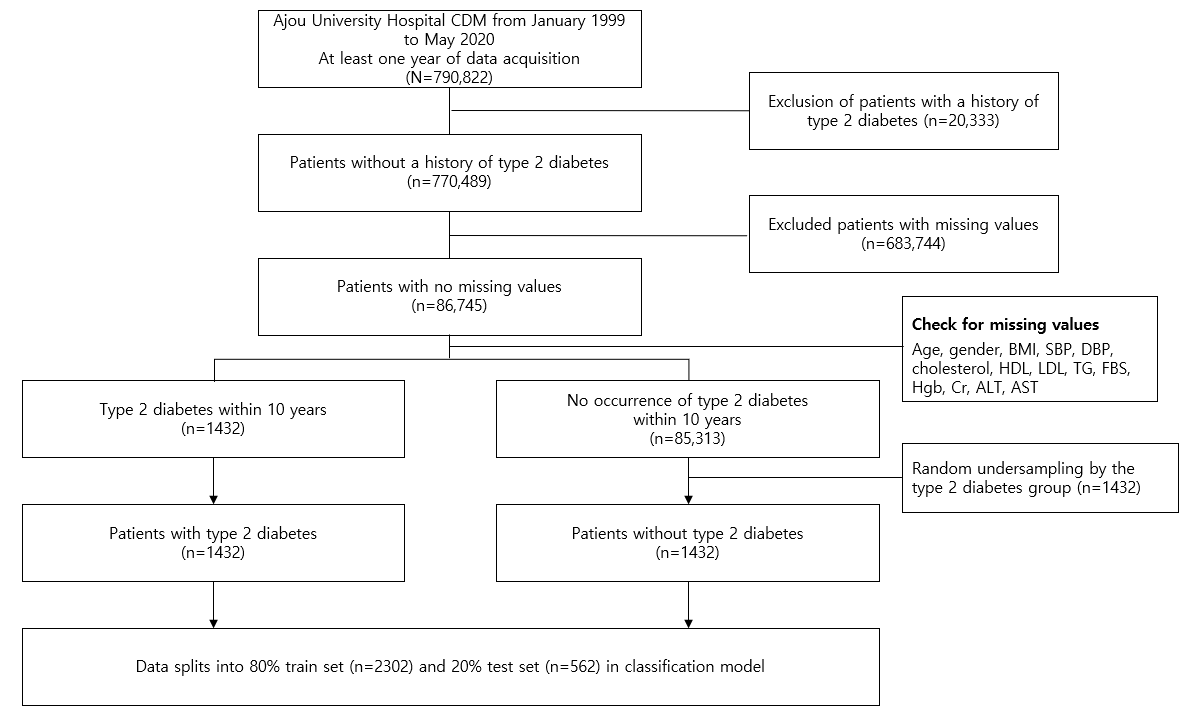
The process of selecting subjects for the type 2 diabetes study. ALT: alanine aminotransferase; AST: aspartate aminotransferase; CDM: common data model; Cr: creatinine; DBP: diastolic blood pressure; FBS: fasting blood glucose; HDL: high-density lipoprotein; Hgb: hemoglobin; LDL: low-density lipoprotein; SBP: systolic blood pressure; TG: triglyceride.

We used the data of 790,822 subjects with at least one year of hospital records among subjects aged ≥20 years who had also visited a tertiary hospital in South Korea (Ajou University Hospital in Suwon) from 1999 to 2020. To predict the risk of developing chronic diseases for the subjects as they age, patients with chronic diseases (type 2 diabetes, high blood pressure, hyperlipidemia, and cardiovascular disease) as the underlying disease were excluded (Figure 1).

### Select Model Variables

The public health checkup is a test for adults aged >18 years in South Korea, and anyone can use it for free. Variables were selected based on the general examination of items from the National Health Insurance Service. A total of 19 variables were included, such as basic information, measurement information, lifestyle information, and history of diseases.

### Data Extraction

The data used in the predictive model were extracted using the Atlas analysis tool (Observational Health Data Sciences and Informatics—a nonprofit consortium that allows researchers to perform design, characterization, and analysis). A cohort for chronic diseases was created through Atlas design for the variables used in the cohort. Concept IDs following the Systematized Nomenclature Of Medicine–Clinical Terms terminology were used, which are mapped to the International Classification of Diseases, 10th Revision code and currently used as a diagnostic name in clinical practice. Systematized Nomenclature Of Medicine–Clinical Terms were developed to meet the various needs and expectations of clinicians around the world, and it is an international standard terminology system used in more than 80 countries, helping to consistently express clinical contents in medical information records. Additionally, concept IDs following the Local Laboratory Result Code terminology, mapped with the managed local code, was used. Local Laboratory Result Code refers to international standard test terms, and medical terms are defined and standardized for the standardization of test codes. Table 1 shows the concept IDs used in the defined cohort group.

The defined cohort group was divided into a disease-occurring group and a nonoccurring group according to the presence or absence of a diagnosed chronic disease within 10 years from the index date (when the criteria for participation in the study were met). Cohort generation and data extraction were performed according to the design criteria shown in Textbox 1.

**Table 1:** Concept ID information.

Variables	Concept ID	Concept name	Type	Vocabulary
Type 2 diabetes	201826	Type 2 diabetes mellitus	Factor	SNOMED-CT^a^
Hypertension	316866	Hypertensive disorder	Factor	SNOMED-CT
Hyperlipidemia	432867	Hyperlipidemia	Factor	SNOMED-CT
Cardiovascular disease	134057	Disorder of cardiovascular system	Factor	SNOMED-CT
BMI	3038553	Body mass index (ratio)	Numeric	LOINC^b^
SBP^c^	3004249	Systolic blood pressure	Numeric	LOINC
DBP^d^	3012888	Diastolic blood pressure	Numeric	LOINC
Total cholesterol	3027114	Cholesterol (mass/volume) in serum or plasma	Numeric	LOINC
HDL^e^	3007070	Cholesterol in high-density lipoprotein (mass/volume) in serum or plasma	Numeric	LOINC
LDL^f^	3028437	Cholesterol in low-density lipoprotein (mass/volume) in serum or plasma	Numeric	LOINC
TG^g^	3022038 3022192	Triglyceride (mass/volume) in serum or plasma	Numeric	LOINC
FBS^h^	3040820 36303387 3037110	Fasting glucose (mass/volume) in serum or plasma	Numeric	LOINC
Hgb^i^	3000963 3027484	Hemoglobin (mass/volume) in blood	Numeric	LOINC
Cr^j^	3016723 3051825	Creatinine (mass/volume) in serum or plasma	Numeric	LOINC
AST^k^	3013721	Aspartate aminotransferase (enzymatic activity/volume) in serum or plasma	Numeric	LOINC
ALT^l^	3006923 46236949	Alanine aminotransferase (enzymatic activity/volume) in serum or plasma	Numeric	LOINC

^a^SNOMED-CT: Systematized Nomenclature Of Medicine–Clinical Terms.

^b^LONIC: Local Laboratory Result Code.

^c^SBP: systolic blood pressure.

^d^DBP: diastolic blood pressure.

^e^HDL: high-density lipoprotein.

^f^LDL: low-density lipoprotein.

^g^TG: triglyceride.

^h^FBS: fasting blood glucose.

^i^Hgb: hemoglobin.

^j^Cr: creatinine.

^k^AST: aspartate aminotransferase.

^l^ALT: alanine aminotransferase.

**Design criteria.**

**Target group**

- Patients who visited the hospital from January 1, 1999, to May 31, 2020
- Patients with data for 180 days before and after the index date
- Patients diagnosed with chronic diseases (type 2 diabetes, hypertension, hyperlipidemia, or cardiovascular disease) within 10 years from the index date

**Comparator group**

- Patients who visited the hospital from January 1, 1999, to May 31, 2020
- Patients with data for 180 days before and after the index date
- Patients who have not been diagnosed with chronic diseases (type 2 diabetes, hypertension, hyperlipidemia, or cardiovascular disease) within 10 years from the index date.

**Exclusion criteria**

- A history of chronic diseases (diabetes, high blood pressure, hyperlipidemia, or cardiovascular disease) for any period before the selection duration
- Missing basic information, examination, and questionnaire items that were selected as essential items in the study for the development of the chronic disease prediction model

### Data Preparation

We used the patient information, medical treatment, and examination data from a tertiary hospital in South Korea for the CDM. If the missing value was a numeric variable, it was replaced with the median of the matching gender for each age group (stratified into 5-year units), and in the case of a categorical variable, it was replaced with the mode of the matching gender for each age group. Since the number of samples between the 2 groups was unbalanced, random undersampling was performed in the nondiabetic group within 10 years to match the size of the diabetic group. Although data balancing can be hidden from the actual prevalence in practice, it ensures model performance for new data by preventing biased learning from highly imbalanced class problems.

### Statistical Analysis

The descriptive statistics of each group (target group and comparator group) are shown in Table 2.

**Table 2:** Descriptive statistics.

Feature		Processed data	Target	Comparator
**Sex, n**				
	Female	1157	586	571
	Male	1691	838	853
Age (years), mean (SD)		47.56 (15.03)	54.94 (12.50)	40.17 (13.60)
BMI, mean (SD)		24.59 (5.68)	25.69 (7.05)	23.50 (3.55)
SBP^a^, mean (SD)		128.1 (16.95)	132.5 (17.49)	123.6 (14.86)
DBP^b^, mean (SD) , mean (SD)		79.29 (11.89)	81.56 (12.04)	77.03 (11.16)
Total cholesterol, mean (SD)		189.4 (39.84)	192.9 (42.40)	185.8 (36.76)
HDL^c^, mean (SD)		51.45 (12.52)	47.55 (10.82)	55.36 (13.01)
LDL^d^, mean (SD)		111.9 (29.57)	114 (28.95)	109.7 (30.13)
TG^e^, mean (SD)		143.1 (55.0)	145.0 (59.06)	115.2 (43.63)
FBS^f^, mean (SD)		116.4 (45.64)	136.7 (54.40)	96.04 (20.31)
Hgb^g^, mean (SD)		14.27 (1.70)	14.18 (1.83)	14.36 (1.56)
Cr^h^, mean (SD)		0.99 (0.68)	1.045 (0.92)	0.93 (0.26)
AST^i^, mean (SD)		27.93 (9.64)	31.71 (8.91)	24.15 (11.20)
ALT^j^, mean (SD)		31.16 (11.89)	37.46 (8.19)	24.87 (14.87)

^a^SBP: systolic blood pressure.

^b^DBP: diastolic blood pressure.

^c^HDL: high-density lipoprotein.

^d^LDL: low-density lipoprotein.

^e^TG: triglyceride.

^f^FBS: fasting blood glucose.

^g^Hgb: hemoglobin.

^h^Cr: creatinine.

^i^AST: aspartate aminotransferase.

^j^ALT: alanine aminotransferase.

### Models

#### Overview

**Figure 2:**
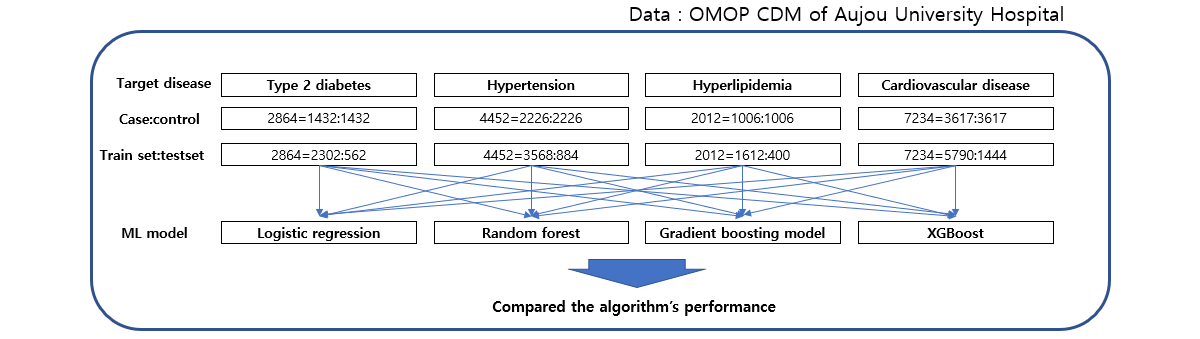
Prediction model flow. CDM: common data model; ML: machine learning; OMOP: Observational Medical Outcomes Partnership; XGBoost: extreme gradient boosting.

In this study, to select the most suitable model for disease prediction, we used the following 4 algorithms: logistic regression (LR), random forest (RF), gradient boosting model (GBM), and extreme gradient boosting (XGBoost). LR using binary classification in the statistics field and the other 3 machine learning algorithms had shown better performance than similar prior research [9]. Afterward, the prediction performance was compared. Model validation was conducted with the same 80% training data and 20% validation data derived from the entire data set. Accuracy, sensitivity, specificity, and AUC were used as model performance indicators. The prediction model flow is shown in Figure 2.

#### LR Algorithm

LR was devised by Cox [10] as a regression model that predicts the probability of the occurrence of an event with respect to a binary dependent variable. Unlike general linear regression analysis, the range of LR is limited to 0-1 because the dependent variable is dichotomous, and the conditional probability of the occurrence of an event also follows a binomial distribution. That is, if the estimated value following the logistic function satisfying the above assumption is less than 0.5, the predicted value is classified as “nonoccurring,” and if it is greater than 0.5, then the predicted value is classified as “occurring.” Although LR was developed in 1970, it is still being used for statistical analysis and predictive research in various fields.

#### RF Algorithm

RF is a tree-based ensemble model capable of both classification and regression and selects the most appropriate forest model by collecting the results of randomly generated independent decision trees [11]. Bagging-based training data inputted to the tree provides model diversity, and the randomness of variable combinations constituting the tree can prevent model noise and the risk of overfitting. The fact that RF is less sensitive to missing values than other algorithms is also an advantage.

#### GBM Algorithm

GBM is a tree-based ensemble model similar to RF, but unlike RF, it creates a tree using a boosting method. The boosting method increases the performance of classification or prediction by sequentially combining several small models [12]. GBM reduces the errors generated by the previous model. Although GBM shows high performance in prediction, it may take a lot of time to fit the model because training requires extensive computation. In recent years, GBM-based algorithms such as LightGBM [13], CatBoost, and XGBoost have been developed to overcome the shortcomings of GBM.

#### XGBoost Algorithm

XGBoost is a representative tree-based ensemble model devised by Chen and Guestrin [14]. It is a machine learning algorithm actively used in prediction and classification research because of its powerful performance and has many advantages such as fast learning due to parallel processing, overfitting regulation, and linkage with other algorithms. Since XGBoost is based on GBM, it optimizes the model by assigning weights using a boosting method, reducing the residual error of the model created with classification and regression tree algorithm–based trees.

### Grid Search

Unlike LR analysis, machine learning algorithms support various parameters (hyperparameters) so that users can optimize the model. Grid search is a technique to find the parameter value when the model has the highest performance by sequentially applying the parameter values set by the user. We optimized the model by applying grid search to the above 3 algorithms (RF, GBM, and XGBoost). Table S1 in Multimedia Appendix 1 presents the parameters and ranges used in the grid search for each algorithm.

## Results

### Model Results

Comparing model performance by chronic disease, the predictive model using XGBoost based on accuracy showed superior performance in all diseases compared to the other 3 models (Table 3).

**Table 3:** Performance comparison of disease prediction models.

Parameter, chronic disease		LR^a^	RF^b^	GBM^c^	XGBoost^d^
**Accuracy**					
	Type 2 Diabetes	0.877	0.8743	0.8743	0.8824
	Hypertension	0.7783	0.793	0.7896	0.8213
	Hyperlipidemia	0.8125	0.82	0.8325	0.8325
	Cardiovascular disease	0.7941	0.8162	0.8235	0.8429
**Sensitivity**					
	Type 2 Diabetes	0.8852	0.8804	0.8684	0.8705
	Hypertension	0.7758	0.7758	0.7783	0.7934
	Hyperlipidemia	0.8141	0.8556	0.8077	0.8182
	Cardiovascular disease	0.8143	0.8644	0.8030	0.8243
**Specificity**					
	Type 2 Diabetes	0.8691	0.8684	0.8804	0.8950
	Hypertension	0.7808	0.7808	0.8019	0.8550
	Hyperlipidemia	0.8109	0.8122	0.8333	0.8482
	Cardiovascular disease	0.8333	0.7792	0.7857	0.8636

^a^LR: logistic regression.

^b^RF: random forest.

^c^GBM: gradient boosting model.

^d^XGBoost: extreme gradient boosting.

### Model Validation Results

Table 4 shows the parameter values of each disease model outputted by the XGBoost grid search.

**Figure 3:**
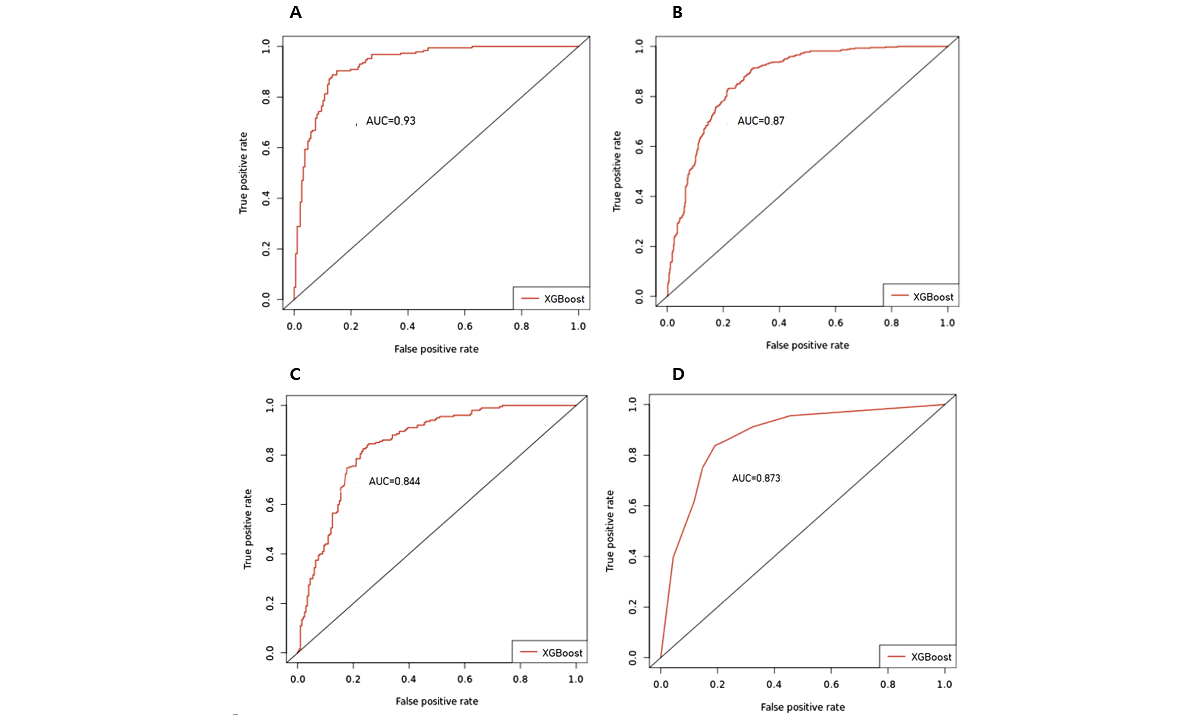
Receiver operating characteristic curves for XGBoost (A) type 2 diabetes model, (B) hypertension model, (C) hyperlipidemia model, and (D) cardiovascular disease model. AUC: area under the curve; XGBoost: extreme gradient boost.

The model evaluation indicators used were accuracy, sensitivity, specificity, and AUC. Over 80% prediction accuracy was achieved for all diseases, with AUC from 0.84 to 0.93. The XGBoost model performance by disease is shown in Table 5 and Figure 3.

**Table 4:** Extreme gradient boosting grid search result.

Target disease	Subsample^a^	Max depth^b^	Min child^c^	Eta^d^
Type 2 diabetes	0.7	7	2	0.1
Hypertension	0.9	3	2	0.01
Hyperlipidemia	1	3	2	0.01
Cardiovascular disease	0.9	3	1	0.01

^a^Subsample: sample’s rate of each tree.

^b^Max depth: maximum depth of Tree.

^c^Min child: minimum sum of weights for all observations needed in the child.

^d^Eta: learning rate.

**Table 5:** Predictive performance by model.

Target disease	Accuracy	Sensitivity	Specificity	AUC^a^
Type 2 diabetes	0.8824	0.8705	0.8950	0.9303
Hypertension	0.8213	0.7934	0.8550	0.8704
Hyperlipidemia	0.8325	0.8182	0.8432	0.8442
Cardiovascular disease	0.8429	0.8243	0.8636	0.8726

^a^AUC: area under the curve.

### Shapley Additive Explanations Model Variable Importance

In open-source program languages (eg, Python and R), the XGBoost package shows model feature importance using its own library. However, small models are more combined and complicated, and the feature importance of small models becomes inconsistent. Therefore, we used the Shapley additive explanations (SHAP) method to represent the model’s feature importance, which had high consistency and accuracy [15]. SHAP’s feature importance used the weighted average of marginal contribution for each feature (Shapley value). It gave the importance of the features and the positive or negative effect of each feature. The formula of Shapley value is a follows:


Contribution of feature_i_ = β_i_x_i_ – E(β_i_x_i_) = β_i_x_i_ – β_i_E(x_i_)

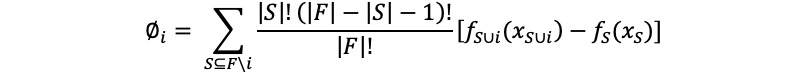



where Ø*_i_* is the Shaley value of data*_i_*, *F* is the full set, *S* is the subsets in total set excluding data*_i_*, 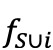 is the contribution of the full set including data*_i_*, and *f_S_ (x_S_)* is the contribution of subsets excluding data*_i_*.

**Figure 4:**
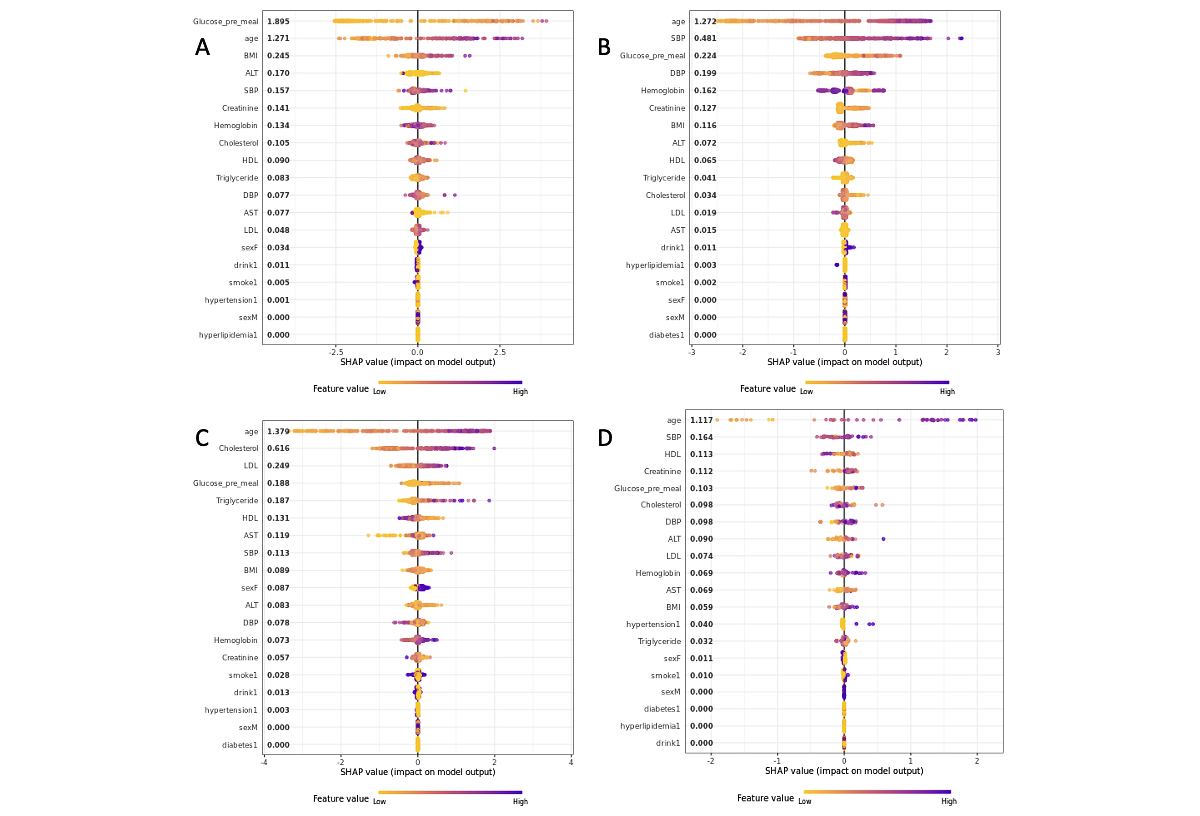
Shapley additive explanations (SHAP) value graph of the fitted model for the importance of (A) type 2 diabetes variables, (B) hypertension variables, (C) hyperlipidemia variables, and (D) cardiovascular disease variables. ALT: alanine aminotransferase; AST: aspartate aminotransferase; DBP: diastolic blood pressure; HDL: high-density lipoprotein; LDL: low-density lipoprotein; SBP: systolic blood pressure.

The SHAP value graph of the fitted model for each disease is presented in Figure 4.

In the case of type 2 diabetes, fasting blood glucose (Shapley value=1.895), age (1.271), and BMI (0.245) influenced the occurrence of diabetes within 10 years [16]. For hypertension, hyperlipidemia (1.272), cardiovascular disease (1.379), and age (1.117) had the greatest influence on disease occurrence. Furthermore, in the case of hyperlipidemia, it was found that among the variables excluding age, total cholesterol (0.616) and low-density lipoprotein (0.249) influenced disease occurrence, in the order presented [17]. In case of cardiovascular disease, systolic blood pressure (0.164) and high-density lipoprotein (0.113) had the second highest influence on disease occurrence. These results are consistent with the results of previous studies that studied the risk factors of the 4 chronic diseases [16,17].

## Discussion

### Principal Findings

This study develops a disease prediction model with more than 80% accuracy by using the 16 National Health Insurance system test variables from the real-world data of a tertiary hospital in South Korea. Our study:

1. Presents the possibility of predicting diseases with universal and useful information on public health examinations,
2. Explains the ability of model prediction results, and
3. Presents the external verification and scalability using other organizations’ CDM.

By observing recent research trends relating to disease prediction models using a CDM, the number of cases focusing on multicenter studies rather than single-center studies is increasing. Lee et al [18] established an artificial intelligence (AI) learning platform for multicenter clinical research focusing on CDM linkage. Using data from Gachon University Gil Hospital to develop a machine learning model that predicts 5-year risk in patients with inflammatory bowel disease who started biologics, Choi et al [19] externally validated the model with CDM data (Ministry of Food and Drug Safety). Johnston et al [20] developed a model to predict whether patients will stop taking antihyperglycemic drugs within 1 to 2 years after laparoscopic metabolic surgery. Using psychiatric patient notes at Ajou University Hospital, Lee et al [21] developed an NLP model that predicts the onset of psychosis in patients by learning, which is a representative case. As such, if the same cohort criterion is applied to multiple institutions in an expanded form along with disease prediction model construction and cross-validation, a more universal and robust model can be developed.

Research is being conducted globally to reduce medical costs by predicting disease occurrence using AI. As the AI industry has gone bigger, AI can reduce costs in providing care and increase the efficiency of medical jobs [22]. In 2019, researchers from the Boston Institute of Technology and Boston Health Center conducted joint research using electronic health records and lifelog big data with AI in an attempt to prevent disease outbreaks and medical fraud. The findings may help reduce hospitalization costs, which account for a substantial portion of US medical expenses [23]. The model developed through this study is expected to evolve into a similar system for South Koreans, by predicting the risk of future disease development and aiding self-health management.

### Comparison With Prior Work

With the recent development of AI and data processing technology, research on disease prediction model development using National Health Insurance Service data [9] or single-institution EMR data [24] is steadily progressing. In this study, we developed chronic disease prediction models with relatively high performance compared to previous papers. A difference between this study and existing work is that the models have been developed using the CDM, so that we can expect improved precision through variable expansion and by simultaneously using multiorganizational data.

### Limitations

A limitation of this study is that it uses a single-institution CDM from a tertiary hospital. Therefore, it cannot ensure generalizability. Additionally, the demographic variables (educational level, residential area, marital status, etc.) are insufficient compared to the health insurance service examination items. They are limited due to the focus on South Korean public checkups; by using more features related with the disease (hemoglobin A1C and biopsy data), the model becomes more accurate. Lastly, the model was trained using the cross-sectional data of patients. If the model is trained using time-series data (eg, the cohort of patients’ information that includes changes of laboratory results as time goes by), it could be much more comprehensive.

### Conclusions

In this study, 4 metabolic chronic diseases were selected, and disease prediction models were developed using the Ajou University Hospital CDM. To obtain a model suitable for disease prediction, the predictive performance of each model for disease occurrence was compared using the LR, GBM, RF, and XGBoost algorithms. The XGBoost model shows the best performance for all diseases. The performance of the XGBoost model was calculated as 0.9303, 0.8704, 0.8442, and 0.8726 AUC standards for type 2 diabetes, hypertension, hyperlipidemia, and cardiovascular disease, respectively. In addition, the importance of the variables was calculated through modeling, and the results are in line with previous clinical studies. We have confirmed that chronic diseases can be predicted, not just using single-institution EMR or public clinical data, but using the CDM in each local hospital.

## Appendix

Multimedia Appendix 1 Model’s hyperparameter range for grid search.

## Abbreviations

AI: artificial intelligence
AUC: area under the curve
CDM: common data model
EMR: electronic medical record
GBM: gradient boosting model
LR: logistic regression
RF: random forest
SHAP: Shapley additive explanations
XGBoost: extreme gradient boosting
