# Supplementary material for: Chronic Disease Prediction Using the Common Data Model: Development Study
Source: JMIR AI. 2022 Dec 22;1(1):e41030. doi: 10.2196/41030 (PMC11041444; doi:10.2196/41030)
Supplement: Multimedia Appendix 1 [file ai_v1i1e41030_app1.docx]

Multimedia Appendix 1. Model’s hyperparameter range for grid search.

| Model | Hyperparameter | Range |
| --- | --- | --- |
| RF | n_estimator | 100, 200, 500, 1000 [default: 100] |
|  | max_depth | 5, 8, 10, 15, 20 [default: none] |
|  | min_samples_leaf | 3, 5, 10, 20, 50 [default: 1] |
|  | min_samples_split | 3, 5, 10 [default: 2] |
| GBM | n.trees | 100, 500, 1000, 5000 [default: 100] |
|  | interaction.depth | 1, 2, 3, 5 [default:1] |
|  | Shrinkage | 0.01, 0.05, 0.1, 0.2, 0.3 [default: 0.1] |
|  | bag.fraction | 0.3, 0.5, 0.7 [default: 0.5] |
| XGBoost | Subsample | 0.7, 0.9, 1 [default: 1] |
|  | Max_depth | 3, 5, 6, 7, 9 [default: 6] |
|  | Min_child | 1, 2, 3 [default: 1] |
|  | eta | 0.01, 0.05, 0.1, 0.3, 0.5 [default: 0.3] |

Note. RF: Random forest GBM: Gradient Boosting Model XGBoost: eXtreme Gradient Boosting
